# Estatinas na Gestação – Novas Recomendações do *Food and Drug Administration*

**DOI:** 10.36660/abc.20220413

**Published:** 2022-07-07

**Authors:** Marcio Sommer Bittencourt

**Affiliations:** 1 University of Pittsburgh Medical Center Division of Cardiology Department of Medicine Pittsburgh EUA Division of Cardiology, Department of Medicine, University of Pittsburgh Medical Center (UPMC), Pittsburgh – EUA

**Keywords:** Inibidores de Hidroximetilglutaril-CoA Redutases, Gestação, Dislipidemias

O *Food and Drug Administration (FDA)*, orgão responsável pelo controle de segurança e eficácia de medicações nos Estados Unidos, tradicionalmente utiliza uma classificação do nível de risco para o uso de medicações durante a gestação. Nesta classificação, as estatinas eram consideradas da categoria X, o que indica um risco demonstrado de anormalidades fetais acima do benefício do seu uso. No entanto, em julho de 2021, o FDA retirou esta recomendação.^1^

Antes da mudança, a recomendação americana era de que as estatinas fossem suspensas desde o início das tentativas de concepção até o término do aleitamento materno. A presente mudança tem embasamento científico adequado. A recomendação prévia de risco fetal associado ao uso de estatinas vem de literatura de estudos experimentais em animais com doses muito mais altas que as utilizadas em humanos.^4^ Já estudos mais recentes e com dados em humanos não demonstram o mesmo risco identificado nos estudos experimentais. Em um estudo observacional recente o uso de estatinas não se associou a maior risco de malformações fetais. No entanto, seu uso associou-se a baixo peso ao nascer e parto prematuro. Ainda, uma metánalise de 5 estudos de coorte publicada recentemente corroborou tais achados.^5^ É importante destacar que o uso de estatinas está associado a maior número de comorbidades que podem levar a baixo peso ao nascer ou parto prematuro. Logo, é possível que estas complicações estejam associadas a comorbidades e não ao uso de estatinas.

Mesmo com o uso seletivo, pesando os riscos e benefícios, esta mudança do FDA tem vários outros desdobramentos. Dentre eles, o principal é facilitar a execução de estudos clínicos com estatinas no periodo pré-concepção, gestacional e durante a amamentação. Além de avaliar o benefício de redução do risco cardiovascular nestes períodos, estudos mais recentes têm investigado outros efeitos potenciais das estatinas. Um estudo publicado neste ano avaliou o impacto da estatina nos resultados de fertilização *in vitro* de pacientes com dislipidemia a infertilidade. Apesar de limitações importantes, o estudo sugere que pravastatina pode melhorar a eficácia do tratamento com fertilizações *in vitro* nesta população. No entanto, estudos adicionais são necessários antes de qualquer implementação clínica.

Da mesma forma, diversos estudos têm avaliado o uso de estatinas na prevenção de pré-eclampsia. O racional destes estudos é que as estatinas poderiam reverter o desbalanço entre angiogenese e antiangiogenese que ocorre antes da apresentação clínica da pré-eclampsia. Apesar de diversos estudos realizados, este efeito das estatinas na pré-eclampsia, ainda é controverso. Estudos menores sugeriram benefício, mas estudos randomizados recentes foram negativos.^6^ Até a realização de estudos adicionais, deve-se considerar o benefício da estatina nestes cenários como incerto.

Apesar de um pequeno passo, a mudança de linguagem e recomendação proposta pelo FDA tem vários desdobramentos clínicos importantes e implicações científicas futuras. Por hora, a simples flexibilização do uso de estatinas durante o período pré-concepção e durante a gestação já terá grande impacto na prática clínica rotineira para mulheres com alto risco cardiovascular e doença aterosclerótica estabelecida, que estejam em idade fértil e desejem engravidar.
