# Supplementary material for: Estatinas na Gestação – Novas Recomendações do Food and Drug Administration
Source: Arq Bras Cardiol. 2022 Jul 7;119(1):1–2. [Article in Portuguese] doi: 10.36660/abc.20220413 (PMC9352124; doi:10.36660/abc.20220413)
Supplement: Translation

# Translation: Statins and Pregnancy – New FDA Recommendations

**Authors:** Marcio Sommer Bittencourt

**Affiliations:** 1 Pittsburgh USA Division of Cardiology, Department of Medicine, University of Pittsburgh Medical Center (UPMC), Pittsburgh – USA

**Keywords:** Hydroxymethylglutaryl-CoA Reductase Inhibitors, Pregnancy, Dyslipidemias

The Food and Drug Administration (FDA), the agency responsible for controlling the safety and efficacy of drugs in the United States, has traditionally adopted a risk classification for the use of drugs during pregnancy. Statins were considered a Category X drug, which indicates that the demonstrated risk of these drugs to cause birth defects surpassed their benefits. However, in July 2021, the FDA withdrew this recommendation.^1^

Before this change, the FDA recommended the discontinuation of statins from conception attempts until the end of breastfeeding. In cases where dyslipidemia is not severe, statin discontinuation may confer no additional risk. However, based on this recommendation, the duration of discontinuation may be substantial. In a study with more than 100 women with familial hypercholesterolemia in Norway and Netherlands, the mean length of time without statins was longer than two years for each pregnancy.^2^ Although two years is a long period, it still may be a conservative estimate. In this study, the mean interval between conception planning and pregnancy was two months. Data in the literature,^2^ however, suggest that the median time to pregnancy is longer, probably around six months for nulliparous women.^3^ Also, in this study,^2^ mean breastfeeding duration was four months. Considering that the World Health Organization recommends exclusive breastfeeding for the first six months of life, and continued breastfeeding along with introducing complementary foods after this period, the real impact of statin discontinuation during the gestation period may be even greater than two years. Also, many women who use statins are older and may desire more than one child, which may prolong the period of time without statins for more than five years during their reproductive period.

In this context, the change of FDA's position may have a substantial impact on reproductive women willing to become pregnant. Also, the new recommendation is rather vague, as it states that statins should be discontinued by many pregnant women, albeit a shared decision-making, weighing risks and benefits, should be the routine strategy to be adopted. The FDA also advocates that the use of statins in the pre-conception period, during which women are trying to get pregnant, is safe. This change may have a particularly strong impact on 10-20% of couples that deal with infertility, and whose pre-conceptional period may be considerably long. Also, the new recommendation has an important impact on women with known cardiovascular disease who are trying to get pregnant, as data in the literature have shown that the benefit on cardiovascular events with the use of statins can be perceived even with the use of statins for periods shorter than four months in high-risk individuals.^1^

Despite limited data on statins in pregnancy, the new recommendation has an adequate scientific basis. The previous guidance based on the fetal risk related to statins has been constructed from data obtained from animal experimental studies with much higher doses than those usually used in humans.^4^ Nevertheless, more recent studies, involving humans, have not demonstrated the same risks identified in experimental studies. A recent observational study has not shown an association of statins with a higher risk of fetal malformations but did show an association with a low birth weight and premature delivery. Similar data were found in a meta-analysis of five recently published cohort studies.^5^ Since the use of statins has been associated with a higher number of comorbidities, it is possible that these complications are associated with comorbidities rather than with statins. Despite that, the potential residual risk of the use of statins makes the risk and benefit discussion the best strategy.

Even with a selective use of statins, considering risks and benefits, by patients with high cardiovascular risk and those with a previous history of cardiovascular disease, the FDA decision may have other ramifications. The main one consists of clinical trials with studies with women in the pre-conception, gestational and breastfeeding periods. These studies go beyond the known cardiovascular effects. One study published this year evaluated the impact of statins on results of *in vitro* fertilization in patients with dyslipidemia and infertility. Despite important limitations, the study suggests that pravastatin improved these results in the studied population. Further studies are needed before clinical implementation.^6^

Similarly, several studies have evaluated the use of statins in preeclampsia prevention. The rational of these studies is that statins could reverse the imbalance of pro- and anti-angiogenesis factors that precedes the clinical manifestations of preeclampsia. Despite numerous studies, the effect of statins, particularly pravastatin, on preeclampsia are still controversial. While small studies have suggested beneficial effects, recent randomized studies have suggested the contrary.^6^ Apparently, additional studies are needed to best define those patients who would most benefit from statins, as well as the best moment to initiate the drug during pregnancy and the most appropriate dose. Until then, the benefit of statins in these scenarios must be considered as uncertain.

It was a small step, but the change in the language and recommendation proposed by the FDA has had important clinical repercussions and future scientific implications. So far, the flexibility in the use of statins in the pre-conception period and during pregnancy will already have a strong effect on routine clinical practice with women of fertile age, at high cardiovascular risk and established atherosclerosis, trying to become pregnant.
